# Neural Correlates of Flight Acceleration in Pigeons: Gamma-Band Activity and Local Functional Network Dynamics in the AId Region

**DOI:** 10.3390/ani15131851

**Published:** 2025-06-23

**Authors:** Suchen Li, Zhuo Tang, Mengmeng Li, Lifang Yang, Zhigang Shang

**Affiliations:** 1 School of Electrical and Information Engineering, Zhengzhou University, Zhengzhou 450001, China; suchenli@gs.zzu.edu.cn (S.L.); tz@gs.zzu.edu.cn (Z.T.); limengmeng@zzu.edu.cn (M.L.); 2 Henan Key Laboratory of Brain Science and Brain-Computer Interface Technology, Zhengzhou 450001, China; 3 The Affiliated Encephalopathy Hospital, Zhengzhou University, Zhumadian 463000, China

**Keywords:** pigeon, flight acceleration, local field potentials, gamma, local functional network

## Abstract

Flight behavior in pigeons is governed by intricate neural mechanisms that regulate movement patterns and flight dynamics. Among various kinematic parameters, flight acceleration provides critical information for the brain to modulate movement intensity, speed, and direction. However, the neural representation mechanisms underlying flight acceleration remain insufficiently understood. To address this, we conducted outdoor free-flight experiments in homing pigeons, during which GPS data, flight posture, and eight-channel local field potentials (LFPs) were synchronously recorded. Our analysis revealed that gamma-band activity in the dorsal intermediate arcopallium (AId) region was more prominent during behaviorally demanding phases of flight. In parallel, local functional network analysis showed that the clustering coefficient of gamma-band activity in the AId followed a nonlinear, U-shaped relationship with flight acceleration—exhibiting the strongest and most widespread connectivity during deceleration, moderate connectivity during acceleration, and the weakest network coupling during steady flight. This pattern likely reflects the increased neural demands associated with flight phase transitions, where greater cognitive and sensorimotor integration is required. Furthermore, using LFP signals from five distinct frequency bands as input, machine learning models were developed to decode flight acceleration, further confirming the role of gamma-band dynamics in motor regulation during natural flight. This study provides the first evidence that gamma-band activity in the avian AId region encodes flight acceleration, offering new insights into the neural representation of motor states in natural flight and implications for bio-inspired flight control systems.

## Simple Summary

Flight acceleration is a key indicator of how motor behavior is dynamically regulated during aerial navigation. In this study, we conducted outdoor free-flight experiments in homing pigeons and analyzed brain activity from the dorsal intermediate arcopallium (AId), a region functionally similar to the mammalian motor cortex. The results showed that both the power spectral density (PSD) proportion and the local Functional Network of gamma-band brain networks varied with flight acceleration, suggesting that neural activity in the AId region encodes changes in flight states. These findings highlight how brain activity adapts to varying motor demands and provide new insights into the neural basis of flight control in birds.

## 1. Introduction

Pigeons—renowned for their ability to navigate complex environments, exhibit home-site fidelity, and demonstrate exceptional spatial orientation—are frequently used as a model in avian neuroscience [1,2,3,4]. Recent advances in decoding movement-related neural signals in pigeons have laid the groundwork for understanding how flight behavior is orchestrated by the brain [5,6,7]. While prior studies have primarily focused on spatial trajectories, flight velocity, and positional encoding, flight acceleration—serving as a dynamic intermediary between multiple kinematic variables—remains comparatively underexplored [8,9,10]. As a key parameter that reflects both the intensity and transition phases of motor output, acceleration offers unique insights into the neural mechanisms underlying precise motor regulation during natural flight.

To clarify the neural mechanisms underlying acceleration encoding, several studies have employed velocity clamp systems or linear-track paradigms in rodents to examine the relationship between the theta oscillations (4–12 Hz) in the hippocampal or medial entorhinal cortex and positive/negative acceleration [10,11]. Complementary investigations using treadmill running, upper-limb reaching, and grasping tasks in nonhuman primates have demonstrated that neurons in the primary motor cortex robustly represent movement acceleration [12,13]. These findings collectively underscore the critical role of acceleration coding in the brain’s multimodal representation of movement and motivate further exploration into its neural substrates.

Due to technical constraints, studies on avian motor control have historically focused on limited two-dimensional representations of movement. However, with recent advancements in lightweight recording and telemetry technologies, it has become increasingly feasible to examine the neural dynamics underlying natural, three-dimensional flight behavior. Compared with the primarily two-dimensional kinematic features of terrestrial locomotion, avian flight imposes far more complex, multidimensional spatiotemporal demands on motor control [14]. Rattenborg et al. [15] recorded EEG from frigatebirds during oceanic flight and found that, in daytime flight, cortical activity was characterized by low-amplitude, high-frequency oscillations. Vyssotski et al. [16] released pigeons 20 km offshore and observed activation across multiple frequency bands when birds traversed salient landmarks such as coastlines, roads, and intersections. Throughout aerial locomotion, acceleration information provides essential feedback for regulating thrust, speed, and heading in response to dynamic environmental conditions. Using a wearable telemetry system, Yang et al. [17] simultaneously recorded neural activity from flying birds and decoded distinct neural features corresponding to different flight modes. Although existing studies have begun to elucidate how motor regions perceive and adjust to acceleration, the dynamic neural encoding of acceleration during high-velocity, outdoor flight remains an open scientific challenge.

Once a bird initiates the decision to fly, it must transmit this signal to its motor output system. Similar to mammals, birds possess two distinct descending pathways for motor control [18]. Within this framework, the dorsal intermediate arcopallium (AId), a subregion of the arcopallium, has been identified as a central hub for regulating motor output during complex flight behaviors [19,20]. Yuan et al. [21] recorded AId neurons in freely behaving juvenile zebra finches and found that a substantial proportion displayed heterogeneous response patterns across multiple motor acts. Benjamin [22] further demonstrated that motor neurons in the songbird AId exhibit rapid and precise firing patterns during fast, skilled movements. These findings highlight the pivotal role of the AId in coordinating avian motor behavior. Given that acceleration is essential for the moment-to-moment adjustment of flight dynamics, the neural representation of acceleration within the AId may be particularly direct and functionally significant.

To elucidate the dynamic neural representation of flight acceleration in natural conditions, we conducted an outdoor free-flight experiment using homing pigeons (*Columba livia domestica*), during which we simultaneously recorded GPS data, flight attitude data, and 8-channel local field potentials from the AId, a region previously identified as functionally analogous to the mammalian motor cortex. Neural activity under varying acceleration conditions during flight was characterized from two complementary perspectives: time–frequency dynamics and functional network topology. In addition, we implemented the rhythm-specific neural decoding of flight acceleration to explore the potential of neural activity in representing motor state transitions. This study reveals, for the first time, that gamma-band activity in the avian AId region encodes flight acceleration, enriching the theoretical understanding of motor state representation during natural flight. It also uncovers a non-linear modulation of AId network topology with acceleration, providing new insights into brain network adaptation during behavioral transitions and supporting the development of bio-inspired control strategies for animal-robot systems.

## 2. Materials and Methods

### 2.1. Outdoor Free-Flight Experiment

To investigate how pigeons dynamically represent acceleration through neural activity during outdoor flight under continuously changing natural conditions—and thereby achieve the precise regulation of their own movement states—we designed an outdoor free-flight experimental paradigm.

The flight experiments were conducted under favorable weather conditions, specifically clear days with good visibility and wind speeds below Beaufort Force 3. Pigeons carrying the recording apparatus were released from a fixed site 2 km from the loft and allowed to fly freely back to the home aviary, during which electrophysiological signals, inertial measurement unit (IMU) data, and GPS coordinates were recorded synchronously. To ensure stable physiological and psychological conditions, each bird was allowed to rest for approximately 20 s prior to release, and data retrieval was performed immediately upon their autonomous return. A maximum of two flights were conducted per day, with an inter-release interval of at least 8 h to prevent fatigue. We defined a single flight release process as an experimental trial. Valid experimental data for each trial included multimodal data from the beginning of the resting state until the first landing after free flight. Across all valid trials, the average flight duration from the release site to the landing site was approximately 139 ± 45 s (mean ± SD). To guarantee the consistency and comparability of neural signals across different flight states, we employed a fixed release point to minimize behavioral variability in flight path, posture adjustments, and take-off dynamics. To guarantee the consistency and comparability of neural signals across different flight states, we employed a fixed release point to minimize behavioral variability in flight path, posture adjustments, and take-off dynamics.

### 2.2. Experimental Animals and Surgery

In this study, six healthy pigeons (P01, P04, P05, P09, P12, and P16) with good weight-bearing flight abilities and stable homing were selected as experimental animals. All experimental and animal care procedures complied with the Guide for Care and Use of Laboratory Animals of Zhengzhou University. The study was approved by the Review Committee of the Experimental Animal Institute of Zhengzhou University.

**Figure 1:**
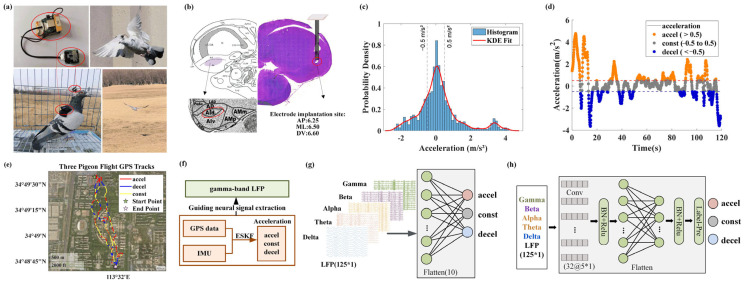
Outdoor free-flight experiment setup and synchronous acquisition of GPS, posture, and local field potential (LFP) data, along with the architecture of the decoder model. (**a**) The pigeon carrying the equipment flies freely outdoors. (**b**) Surgical implantation of the recording electrode array and postoperative histological verification of the implantation site. (**c**) Acceleration distribution and kernel density estimation of a representative pigeon flight experiment. (**d**) IMU data from a representative flight experiment, with acceleration segmented into different states for further analysis. (**e**) GPS trajectories from three outdoor flight experiments in pigeons. (**f**) Block diagram illustrating the integration and processing of GPS data, flight posture information, and LFPs in the decoding framework. (**g**) The architecture of the DNN-based decoding model (**h**) The architecture of the CNN-based decoding model.

Each pigeon was initially anesthetized via the intraperitoneal injection of sodium pentobarbital (0.15 mL/100 g) and secured in the prone position on a custom stereotaxic apparatus until deep anesthesia was confirmed. After infiltrating the scalp with lidocaine for local anesthesia, a midline incision was made to expose the skull. Stereotaxic coordinates for the AId were determined based on the pigeon brain atlas (anteroposterior [AP], 6.25 mm; mediolateral [ML], 6.5 mm; dorsoventral [DV], 6.6 mm). A craniotomy was performed over the target site to reveal the dura, and an eight-channel microelectrode array (2 × 4 configuration; platinum–iridium alloy; 50 µm diameter; 300 µm inter-tip spacing) was implanted. The array was secured with dental cement, and the incision was closed with sutures. Following a seven-day postoperative recovery period, all birds demonstrated homing behavior indistinguishable from pre-surgical performance. Upon the completion of the free-flight data acquisition, the histological verification of electrode placement was carried out via electrolytic lesion, confirming that all electrode tips resided within the intended AId boundaries (Figure 1b).

### 2.3. GPS, Posture and Local Field Potentials Data Synchronously Acquisition and Preprocessing

During the experiment, a custom-designed, wearable multimodal data recorder developed by our laboratory for avian field applications was used (Figure 1a). The system comprised three functional units (Table 1). The neural signal recording unit employed the ADS1299 chip to record eight channels of neural signals with a resolution of 0.1 µV at a sampling rate of 1000 Hz. The posture information recording unit used an IMU mounted at the electrode interface on the pigeon’s head—an area with relatively limited movement—to record tri-axial acceleration and angular velocity at 200 Hz. The flight trajectory recording unit consisted of a GPS module (sampling rate: 10 Hz; positional accuracy: 2.5 m CEP), which was mounted on the pigeon’s back along with a 3.7 V lithium-ion battery to enhance satellite reception, improve positioning accuracy, and minimize interference with natural flight posture.

Adhering to a lightweight design, the complete system weighs 14.3 g—approximately 3% of a pigeon’s body weight. The head-mounted neural recording unit and IMU are connected to the back-mounted GPS via flexible cables to maintain clock synchronization. All sensor modules were controlled and data-streamed by a STM32 microcontroller unit (STMicroelectronics, Geneva, Switzerland), which served as the central processing platform to coordinate multi-sensor data acquisition, manage sampling synchronization, and store the recorded data onto a TransFlash memory card. The STM32 microcontroller unit was programmed with multi-task scheduling to ensure time-aligned recording across the neural, inertial, and GPS subsystems despite their differing sampling rates.

To address the challenge of accurately estimating flight acceleration—caused by the limited spatiotemporal resolution of GPS and the coupling of gravitational and body-motion components in IMU-derived acceleration signals—this study employed an Extended State Kalman Filter (ESKF) framework. Within a nonlinear state-space model, the ESKF enables the optimal fusion of IMU and GPS data to jointly estimate a pigeon’s attitude, velocity, and position, from which flight acceleration was subsequently derived. The filter adopted a 15-dimensional error state vector comprising position errors (3 dimensions), velocity errors (3 dimensions), attitude errors (3 dimensions), gyroscope biases (3 dimensions), and accelerometer biases (3 dimensions), all defined in the local-level navigation frame (East–North–Up). The measurement vector was constructed from GPS-provided position information in this frame, resulting in a 3-dimensional observation vector. State prediction and update followed the standard ESKF equations:

$${\hat{X}}_{k|k-1}=\Phi {\hat{X}}_{k-1}$$

$${\hat{X}}_{k}={\hat{X}}_{k|k-1}+K_{k}\left(z_{k}-H{\hat{X}}_{k|k-1}\right)$$

$$K_{k}=P_{k|k-1}H^{T}{\left(HP_{k|k-1}H^{T}+R\right)}^{-1}$$

$$P_{k|k-1}=\Phi P_{k-1}\Phi^{T}+Q$$

$$P_{k}=\left(I-K_{k}H\right)P_{k|k-1}{\left(I-K_{k}H\right)}^{T}+K_{k}RK_{k}^{T}$$

where $\hat{X}$ denotes the estimated state vector; Φ is the state transition matrix; $K_{k}$ is the Kalman gain; and H, P, Q, and R represent the observation matrix, error covariance matrix, process noise covariance matrix, and observation noise covariance matrix, respectively. The estimated flight acceleration was subsequently used to guide neural signal feature extraction, ensuring that the analysis of neural activity was aligned with the dynamic changes in the bird’s motion state.

The experimental duration was limited as a result of natural environmental interference and biocompatibility constraints. To ensure the reliability of the analysis, flight data from each pigeon were screened based on two criteria: the integrity of multimodal data, where trials with missing GPS or IMU signals due to equipment failure or environmental factors (e.g., signal loss or obstruction) were excluded, and the stability of flight behavior, where trials involving abnormal actions—such as prolonged pausing, frequent interruptions, or significant deviations from the flight path—were removed.

During natural flight, pigeons often exhibit slight fluctuations in instantaneous speed due to periodic wing flapping, airflow disturbances, and subtle postural adjustments, which typically do not reflect meaningful behavioral transitions. The statistical analysis of acceleration data across multiple free-flight trials revealed that acceleration values followed a near-normal distribution centered around 0 m/s^2^. Figure 1c illustrates the acceleration profile obtained from a single representative outdoor flight trial. Based on this, changes within the range of ±0.5 m/s^2^ were defined as a “behaviorally stable zone” representing non-behaviorally driven perturbations and classified as steady flight. Combined with a flight speed threshold of >2 m/s [23,24], flight dynamics were classified into three distinct states: an acceleration state, where speed exceeds 2 m/s and acceleration is greater than 0.5 m/s^2^; a steady state, where speed remains above 2 m/s and acceleration stays within ±0.5 m/s^2^; and a deceleration state, where speed exceeds 2 m/s and acceleration is below –0.5 m/s^2^ (Figure 1d,e).

Due to the weak nature of neural signals, they are highly susceptible to noise, particularly baseline drift and wingbeat artifacts. A bandpass filter (0.5–200 Hz) was applied to eliminate baseline drift. Since the frequency spectrum of wingbeat artifacts substantially overlaps with that of neural activity (6–10 Hz) [25], conventional low-pass filtering may inadvertently suppress informative components of the signal. To address this, Variational Mode Decomposition (VMD) was employed to isolate intrinsic mode functions. Given that wing motion primarily affects vertical (z-axis) acceleration, the similarity between each mode and the z-axis acceleration was calculated to identify noise-contaminated components. These components were then attenuated by reducing their weights, thereby preserving relevant neural information while suppressing wing-related artifacts.

### 2.4. Neural Signal Analysis

During free flight, acceleration dynamics may be reflected in frequency-specific neural oscillations within the AId brain region. To reveal the rhythmic and spectral characteristics of neural signals, the Welch method was used to estimate their power spectral density (PSD) and calculate the power proportion across different frequency bands. The Continuous Wavelet Transform (CWT) was employed to investigate the time–frequency characteristics of neural signals in the AId region under different flight states. The CWT is defined as follows:

(1) $$X_{\omega }\left(a,b\right)=\frac{1}{{\left|a\right|}^{1/2}}\int_{-\infty }^{+\infty }x\left(t\right)\hat{\varphi }\left(\frac{t-b}{a}\right)dt$$

where $\hat{\varphi }$ denotes the mother wavelet function in the frequency domain, a is the scale parameter, b is the translation parameter, $x\left(t\right)$ is the time-series signal, and $X_{\omega }\left(a,b\right)$ represents the wavelet coefficients.

Furthermore, multi-channel local functional network analysis captures inter-regional interactions and information transfer [26,27]. After initially determining the characteristic frequency bands of the neural signals in the resting and flight states of pigeons, the neural signals were intercepted using the sliding window method, and the dynamic functional brain network was constructed based on inter-channel coherence. A higher coherence between two signals tends to represent more reliable information transmission, and the calculation formula is as follows:

(2) $$C_{xy}\left(f\right)=\frac{{\left|G_{xy}\left(f\right)\right|}^{2}}{G_{xx}\left(f\right)G_{yy}\left(f\right)}$$

where $G_{xy}$ is the cross-spectral density between the two signals X(t) and Y(t). Furthermore, to quantitatively measure the closeness of the functional network connections, the topological characterization of the functional brain networks in both the resting and flight states was performed using clustering coefficients:

(3) $${CC}_{i}=\frac{2E_{i}}{d_{i}(d_{i}-1)}$$

where $E_{i}$ represents the number of “edges” actually formed by the other nodes connected to node i and $d_{i}$ represents the degree of node i.

### 2.5. Statistical Test

For group comparisons, a one-way analysis of variance (ANOVA) was applied when the data passed the normality test; otherwise, the Kruskal–Wallis test was used as a non-parametric alternative. The significance level was set at 5%. In the figures, “*” indicates p < 0.05, “**” indicates p < 0.01, and “***” indicates p < 0.001.

### 2.6. Decoding Flight States Using Machine Learning Models

To decode flight motor states from neural activity, machine learning-based models were constructed using LFP signals from five distinct frequency bands as input. Specifically, three algorithmic frameworks were employed: Support Vector Machines (SVMs), Deep Neural Networks (DNNs), and Convolutional Neural Networks (CNNs). These data-driven models aim to automatically extract key features related to flight states from raw neural signals and enable the accurate classification of different flight conditions. The architectures of the decoding models are illustrated in the Figure 1f,g. To reduce the performance fluctuations caused by random initial data partitioning, a 10-fold cross-validation repeated 10 times was employed.

## 3. Results

To investigate the neural signal characteristics of the pigeon AId brain area during different flight acceleration states, the modal components contaminated by wing noise were first identified and down-weighted to reduce their influence. Based on the denoised signals, features were extracted in the single-channel time–frequency domain, and characteristic neural frequency bands corresponding to acceleration states were identified. Subsequently, a multi-channel dynamic functional brain network based on coherence was constructed, and topological analysis from a graph-theoretic perspective was performed to reveal the network structure and its dynamic changes across channels under different acceleration conditions during flight.

### 3.1. Results of Wingbeat Artifact Removal

**Figure 2:**
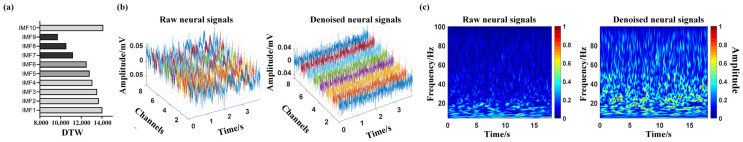
Results of wingbeat artifact removal. (**a**) Correlation measurement of each IMF with wingbeat noise. (**b**) Time–frequency representations of the original and denoised neural signals. (**c**) Time domain plot of the original and denoised neural signals.

To ensure the validity and robustness of the subsequent analyses, all trials from each individual pigeon were subjected to strict quality control (Table 2). The wingbeat artifact is particularly challenging to correct, primarily because artifacts exhibit higher amplitudes than neural signals, possess a broad spectral distribution, and display a variable wingbeat frequency. We performed a 10-layer VMD decomposition of the neural signals during flight. To filter out components disturbed by wing noise, we computed the Dynamic Time Warping (DTW), which is an algorithm that measures the similarity between two time series by distance, of each component with respect to the z-axis acceleration. The more similar two series are, the smaller the value calculated by the DTW. Taking one flight trial as an example, its correlation was calculated (Figure 2a). IMF7, IMF8, and IMF9 were clearly more correlated with wing noise, so we assigned lower weights to them, thus weakening the effect of wing noise on the neural signal.

The original and denoised neural signals are illustrated in both time and time–frequency domains (Figure 2b,c). It can be seen that the noise present in the original signals across multiple octaves has been removed and the energy information previously submerged by motion artifacts has been brought to the forefront, suggesting that the denoising method achieved good results.

### 3.2. Time–Frequency Domain Characterization of AId Neural Signals During Flight Acceleration

**Figure 3:**
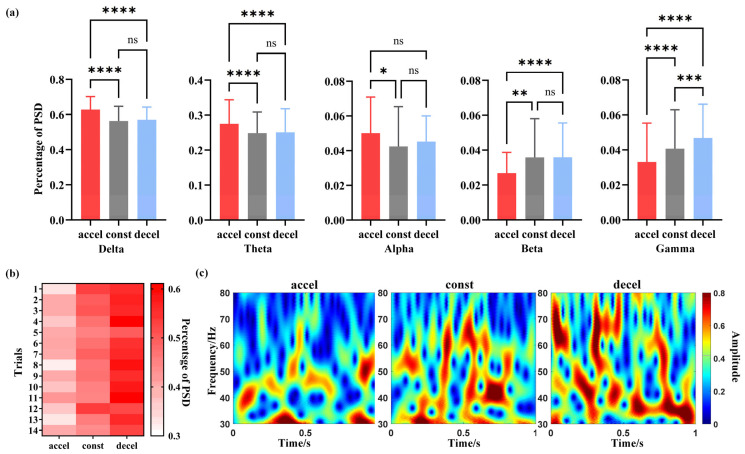
Time–Frequency Domain Characterization of AId Neural Signals During Flight Acceleration. (**a**) Statistical analysis of the PSD proportions of AId neural signals across different flight states. (**b**) Proportion of PSD in the gamma band across valid flight trials under different acceleration states. (**c**) Time–frequency characteristics of gamma-band neural activity in the AId under different flight states. “*” indicates p < 0.05, “**” indicates p < 0.01, “***” indicates p < 0.001, “****” indicates p < 0.0001, ns-not significant.

Neuroscientific research has demonstrated that neuronal firing patterns exhibit distinct rhythmic characteristics across specific frequency bands, each of which is closely linked to particular behavioral states. To explore the neural encoding mechanisms underlying flight motion states, statistical analyses were conducted on the PSD proportions across different frequency bands of AId neural signals under varying acceleration conditions (Figure 3a). Data were collected from all valid flight trials across multiple pigeons (Figure 3b). The results revealed that the PSD proportion in the gamma band significantly differed across acceleration states. Specifically, as the flight state transitioned from deceleration to steady flight and then to acceleration—corresponding to a dynamic shift in acceleration values from negative to zero and then to positive—the gamma-band PSD proportion exhibited a decreasing trend with increasing acceleration.

To visually examine the energy distribution characteristics of gamma-band neural activity in the AId region, time–frequency energy analysis was performed using continuous CWT under different flight acceleration states (Figure 3c). Color indicates energy magnitude, ranging from blue (low) to red (high), and reflects the intensity of neural activity at specific time–frequency points. Warmer colors (closer to red) indicate higher energy levels and thus stronger neural activity within a given frequency band at a particular time point, while cooler colors (closer to blue) represent lower energy levels and weaker neural responses. During the deceleration state, the gamma-band energy distribution was markedly higher than that observed during constant-speed or accelerating flight, exhibiting a sustained high-energy pattern. This suggests heightened neural activation in the AId region under deceleration. These findings further support the results of the power spectral analysis and reveal distinct time–frequency neural response patterns associated with different flight acceleration states.

**Figure 4:**
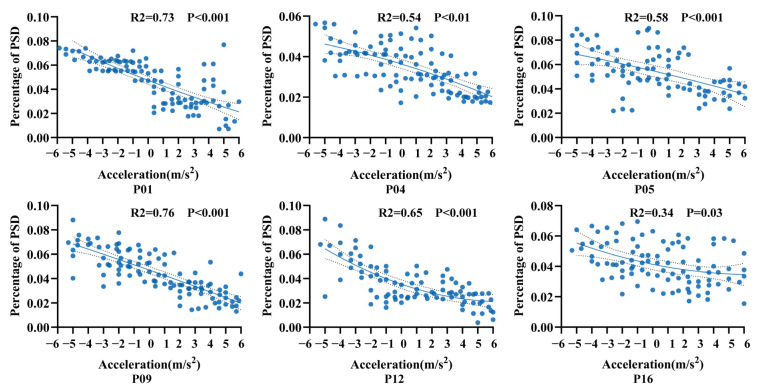
Changes in gamma-band PSD proportion in the AId region across different flight acceleration levels in pigeons. Each subplot shows the scatter distribution and fitted regression curve for an individual subject, revealing a decreasing trend in gamma-band activity with increasing acceleration.

To further investigate the relationship between neural activity and flight acceleration, the acceleration range of –6 m/s^2^ to 6 m/s^2^ was subdivided into finer intervals. For each interval, the mean gamma-band PSD proportion was calculated based on valid flight trials. Polynomial regression was then applied to fit the relationship between acceleration and gamma-band PSD proportion. The results revealed an overall decreasing trend in gamma-band PSD proportion as acceleration increased from negative (deceleration) to positive values (acceleration) (Figure 4). Each scatter point represents the gamma-band PSD proportion at a given acceleration level; the solid line indicates the fitted trend, while the dashed lines denote the 95% confidence interval. These findings suggest that gamma-band PSD variations in the AId region reflect flight acceleration magnitude, implying that this region may be involved in encoding acceleration-related information during natural flight.

### 3.3. Functional Network Analysis of AId Neural Activity Under Flight Acceleration States

**Figure 5:**
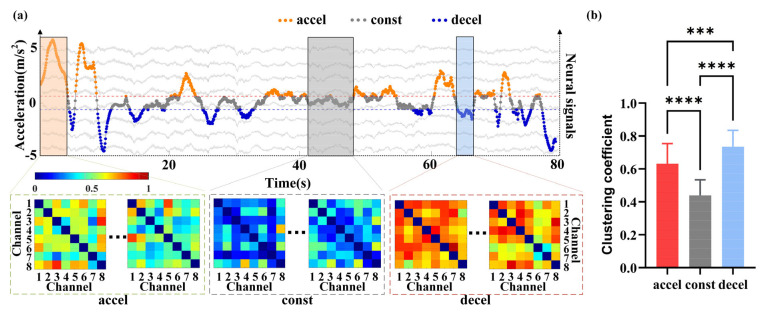
Changes in gamma-band functional connectivity of the AId region across different flight states in pigeons. (**a**) Coherence matrix heatmaps of gamma-band LFP signals across different flight states from a single flight trial. (**b**) Statistical analysis of the clustering coefficients of gamma-band functional networks under different flight states. “***” indicates p < 0.001, “****” indicates p < 0.0001.

Building upon the preceding analysis, a local functional network of the AId region was constructed, and the role of gamma-band neural activity in encoding flight acceleration was further investigated from the perspective of network topological properties. In all valid flight trials, neural signal segments corresponding to different acceleration states were extracted. A sliding window approach was used to calculate the coherence coefficients of gamma-band LFP signals across channels, generating functional connectivity matrices visualized as heatmaps. The clustering coefficient of each network was then quantitatively analyzed to evaluate topological differences under varying flight acceleration conditions (Figure 5a). The heatmap color scale ranges from 0 to 1, where dark blue indicates no connectivity and dark red represents strong coherence. The results reveal that the AId region exhibits relatively strong functional connectivity during both acceleration and deceleration states, with the most robust network observed during deceleration. In contrast, during constant-speed flight, functional connectivity is significantly reduced, showing the sparsest network structure. This state-specific pattern may reflect the neural representation of behavioral demands. Deceleration often serves as a critical transition phase for maneuvering behaviors such as landing, turning, or hovering, requiring pigeons to integrate multisensory inputs—including visual, vestibular, and spatial information. Consequently, the AId region may become more actively involved in perceptual integration and motor regulation, resulting in denser functional connectivity. In contrast, constant-speed flight represents a stable, energy-efficient state with reduced sensory and motor demands. The AId’s functional network in this condition tends to adopt a low-energy, locally connected pattern accompanied by a lower clustering coefficient (Figure 5b). This observation is consistent with the “neural efficiency hypothesis”, which posits a negative correlation between energy consumption and functional connectivity.

**Figure 6:**
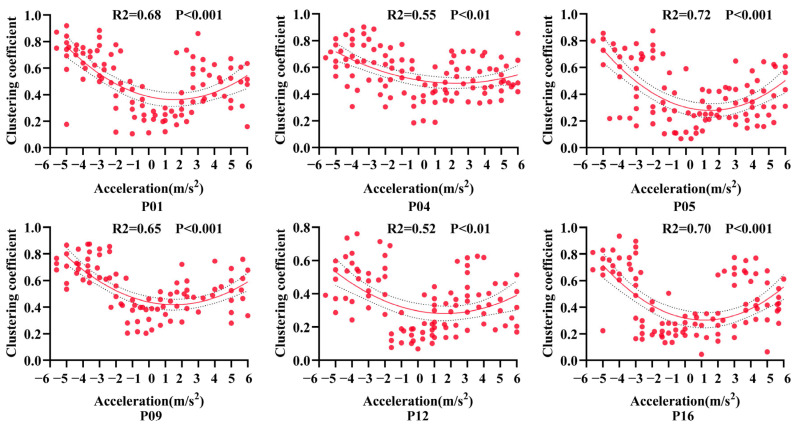
Dynamic changes in the topological properties of gamma-band functional networks in the AId region under different flight acceleration levels in pigeons. Each subplot presents the relationship between acceleration and the clustering coefficient, with second-order polynomial regression indicating non-linear modulation patterns.

To further investigate whether gamma-band functional connectivity in the AId region can dynamically reflect the magnitude of flight acceleration, in this section, we analyzed the topological properties of the network by computing the average clustering coefficient of gamma-band functional networks across multiple trials under different acceleration conditions. A polynomial regression was then applied to assess the trend of clustering coefficient variation with respect to acceleration (Figure 6). Each scatter point represents the average clustering coefficient of the AId network at a given acceleration level; the solid line denotes the fitted trend, and the dashed lines indicate the 95% confidence interval. The results revealed a consistent “U-shaped” relationship between the clustering coefficient and acceleration across all subjects: the clustering coefficient was highest during deceleration, followed by acceleration, and lowest around zero acceleration, corresponding to the constant-speed flight phase. These findings suggest that the dynamic topological properties of the gamma-band (30–80 Hz) LFP-based functional networks in the AId region encode flight acceleration levels. Specifically, during phases of high behavioral demand—such as deceleration and acceleration, which involve rapid motor adjustments and increased cognitive load—the AId region exhibits enhanced local connectivity, reflecting greater functional integration and regulation by neuronal populations under cognitively demanding conditions.

### 3.4. Gamma-Band Specificity in Neural Decoding of Flight States

Based on the above results, the dynamic characteristics of gamma-band LFP signals in the AId region of pigeons were found to be closely associated with flight motor states. Building on this foundation, machine learning-based decoding models were developed using LFP signals from five distinct frequency bands as input, with the goal of further validating the discriminative role of the gamma band. The stability and effectiveness of each model were quantitatively evaluated by computing the mean and standard deviation of performance metrics across all folds of 10-fold cross-validation, which was repeated 10 times to ensure robustness. (Table 3). The chance level for classification is indicated as 33.3%, corresponding to the three flight states in the decoding task. Bold values highlight the highest decoding accuracy within each frequency band. The *p*-values of the statistical comparisons have been added to the table legend to provide clarity on the significance testing procedure.

In all decoding tasks across frequency bands, the gamma band consistently yielded a significantly higher classification accuracy than other bands, further confirming its specificity in representing the neural signatures of flight states. Among the evaluated models, the CNN achieved the highest accuracy across all frequency bands. This suggests that CNNs are capable of automatically learning and extracting discriminative features related to flight states directly from raw neural signals, thereby avoiding the potential subjectivity and bias introduced by traditional feature engineering approaches. These decoding results not only validate gamma-band activity as an effective neural representation of motor states but also establish a quantitative, end-to-end mapping between raw brain signals and behavioral outputs. This provides methodological support for uncovering the neural encoding strategies underlying flight control in birds.

## 4. Discussion

In natural environments, pigeons must continuously adapt to complex and rapidly changing external conditions during flight. Unlike constant-speed flight, which typically represents a stable and energy-efficient cruising state, acceleration and deceleration phases involve rapid changes in motor output and often coincide with behaviorally significant events such as takeoff, landing, turning, and obstacle avoidance. This study investigated the neural representation of flight acceleration in pigeons by analyzing gamma-band LFP dynamics and local functional network properties in the dorsal intermediate arcopallium (AId). The findings provide novel insights into how motor states during natural flight are encoded and decoded from mesoscopic neural signals.

The AId, a key avian forebrain region, receives inputs from numerous brain areas and serves as a major source of descending sensory and motor projections [28]. Within this structure, the AId contains motor-related neurons that are capable of precise and rapid firing during the execution of fast and complex motor skills. Our results provide converging evidence that gamma-band neural oscillations in the AId region are modulated by flight acceleration and may play a role in encoding motion dynamics during natural flight. This finding is consistent with a substantial body of research demonstrating that gamma oscillations are present and modulated in the motor cortex during the planning and execution of movement [29,30]. During motor execution, gamma-band activity typically increases—a phenomenon referred to as movement-related gamma synchronization [31,32]. Such synchronization is believed to reflect the coordinated engagement of local neural assemblies involved in precise motor control, and it may facilitate the integration of sensory feedback with ongoing motor output. The modulation of gamma-band power and connectivity observed in the AId region during different flight acceleration states suggests a similar mechanism may be at work in avian motor systems, supporting rapid adjustments and behavioral transitions during natural flight.

The observed modulation of gamma-band power and functional connectivity with flight acceleration indicates that this frequency band reflects the transitions of behavioral states during pigeon flight. Notably, the superior classification performance of the gamma band across all decoding models further underscores its specificity in representing motor state transitions. While these findings are consistent with previous reports linking gamma oscillations to behavioral complexity, attentional engagement, and movement coordination in mammals [33,34], the functional role of gamma-band activity in the avian brain remains to be fully elucidated and requires further avian-specific investigations to establish direct parallels.

Time–frequency analysis revealed that gamma-band activity in the AId region decreased as acceleration increased from negative to positive values, suggesting heightened engagement during behaviorally demanding phases, particularly deceleration. This was further supported by the CWT analysis, which showed sustained high-energy gamma-band activity during deceleration compared with constant-speed and acceleration phases. These observations may reflect the increased neural processing demands required for precise control and adaptive adjustment during rapid deceleration events, such as obstacle avoidance or landing preparation [35]. From a network topology perspective, gamma-band functional connectivity—quantified via inter-channel coherence—also varied systematically with flight acceleration. Deceleration was associated with the strongest and most widespread network connectivity, followed by acceleration, while steady-speed flight exhibited the sparsest connectivity patterns. The U-shaped relationship observed in the clustering coefficient values reinforces this interpretation, indicating that local network integration is enhanced during transitional flight phases and attenuated during stable cruising. Taken together, these findings support the view that gamma-band dynamics in the AId region reflect the neural orchestration of behavioral state transitions. This work contributes to a deeper understanding of how animals flexibly regulate motor behavior in response to environmental demands and offers valuable implications for the development of adaptive, bio-inspired flight control systems.

Despite the insights gained from this study, several limitations should be acknowledged. First, the analysis exclusively focused on LFP recordings from the AId region, whereas flight acceleration in natural conditions arises from the integration of multiple sensory modalities, including visual, vestibular, and proprioceptive inputs [36,37,38]. Therefore, it cannot be completely ruled out that visual information—particularly dynamic visual cues such as optic flow that systematically vary with flight acceleration—may indirectly influence AId activity via multisensory integration processes. A more comprehensive understanding of sensory–motor interactions during flight will require simultaneous recordings from both motor and visual processing regions to directly disentangle these influences. Second, while the primary wingbeat frequency typically falls within the 6–10 Hz range, higher-order harmonics and nonlinear cross-frequency interactions may still contaminate higher frequency bands, including the gamma range. Although the applied VMD-based method is effective in reducing direct wingbeat interference and enhancing the signal-to-noise ratio of neural activity, the possibility of residual movement-related components cannot be entirely excluded. Finally, this study solely relied on LFP signals, which reflect population-level activity but do not capture the precise spiking dynamics of individual neurons [39]. Incorporating spike-based analyses in future work may reveal complementary encoding mechanisms and finer temporal structures underlying flight-related motor processing.

## 5. Conclusions

This study systematically analyzed the time–frequency characteristics and functional network topology of gamma-band neural activity in the AId region of pigeons during free flight under varying acceleration conditions. The results demonstrated that both the PSD proportion and the clustering coefficient of gamma-band functional networks systematically changed with flight acceleration, indicating that neural activity in the AId region dynamically encodes flight-related motor states. Notably, during acceleration and deceleration—phases typically associated with higher cognitive and sensorimotor demands—the AId region exhibited increased gamma-band activity and denser network connectivity, suggesting its critical role in integrating sensory inputs and executing motor adjustments during flight control. In addition, decoding models based on raw gamma-band LFP signals effectively classified flight acceleration states, providing further support for the representational value of gamma oscillations in motor state differentiation. These findings provide empirical evidence for how avian brain regions process complex motor information in natural environments, and they lay a foundation for future research on brain–behavior relationships and the neural mechanisms underlying biologically inspired flight control systems.

## Figures and Tables

**Table 1:** Sensor specifications of the wearable multimodal data recording system for avian outdoor flight.

Model	Function	Sampling Rate	Mounting Position	Weight
ADS1299	Acquisition of 8-channel neural signals	1000 Hz	Head-mounted	1.26 g
MPU6050	Acquisition of posture data (IMU)	200 Hz	Head-mounted	1.27 g
ATGM336H-5N	Acquisition of GPS data	10 Hz	Back-mounted	1.77 g

**Table 2:** Statistical summary of valid experimental trials.

ID	Experimental Date	Total Trials	Valid Trials
P01	20240507–20240611	41	24
P04	20240812–20240915	33	19
P05	20241107–20241126	27	14
P09	20241206–20250110	38	20
P12	20250209–20250315	39	18
P16	20250209–20250315	36	18

**Table 3:** Decoding accuracy of flight acceleration across different frequency bands.

Model	Theta	Delta	Alpha	Beta	Gamma
SVM	41.77 ± 6.64	43.67 ± 7.35	44.76 ± 7.29	57.61 ± 8.52	**73.49 ± 5.31**
DNN	50.38 ± 6.08	58.53 ± 8.86	64.16 ± 6.63	78.15 ± 8.85	**83.68 ± 6.29**
CNN	64.02 ± 7.25	67.54 ± 9.12	73.58 ± 8.10	83.61 ± 7.33	**89.60 ± 5.05**

## Data Availability

The datasets analyzed in the current study are available from the corresponding author on reasonable request.
